# Resolution of severe SAM-related mitral regurgitation in sigmoid septum by MitraClip in a 95-year-old woman

**DOI:** 10.1007/s12928-025-01219-1

**Published:** 2025-11-28

**Authors:** Ryotaro Yamada, Takao Morikawa, Akihiro Hayashida, Koichiro Imai, Yoji Neishi, Shiro Uemura

**Affiliations:** 1 Department of Cardiology, Kawasaki Medical School, 577 Matsushima Kurashiki, Kurashiki, 701-0192 Japan; 2 Division of Cardiology, The Sakakibara Heart Institute of Okayama, Okayama, Japan

**Keywords:** MitraClip, SAM MR

A 95-year-old woman was admitted with acute decompensated heart failure and progressive pleural effusion. Despite optimal medical therapy (azilsartan 20 mg, amlodipine 5 mg, azosemide 30 mg, bisoprolol 5 mg), heart failure control remained difficult. Baseline transthoracic echocardiography (Fig. 1A) demonstrated a prominent sigmoid septum with systolic anterior motion (SAM) of the anterior mitral leaflet. With Valsalva provocation, MR worsened to severe and LVOT velocity increased to > 4 m/s (see Supplementary Fig. 1A). Transesophageal echocardiography (Fig. 1B) similarly showed SAM with trivial MR and an LVOT velocity of 2.7 m/s at rest, which similarly increased to > 6 m/s with handgrip provocation, accompanied by severe MR (see Supplementary Fig. 1B, 1 C).

**Fig. 1:**
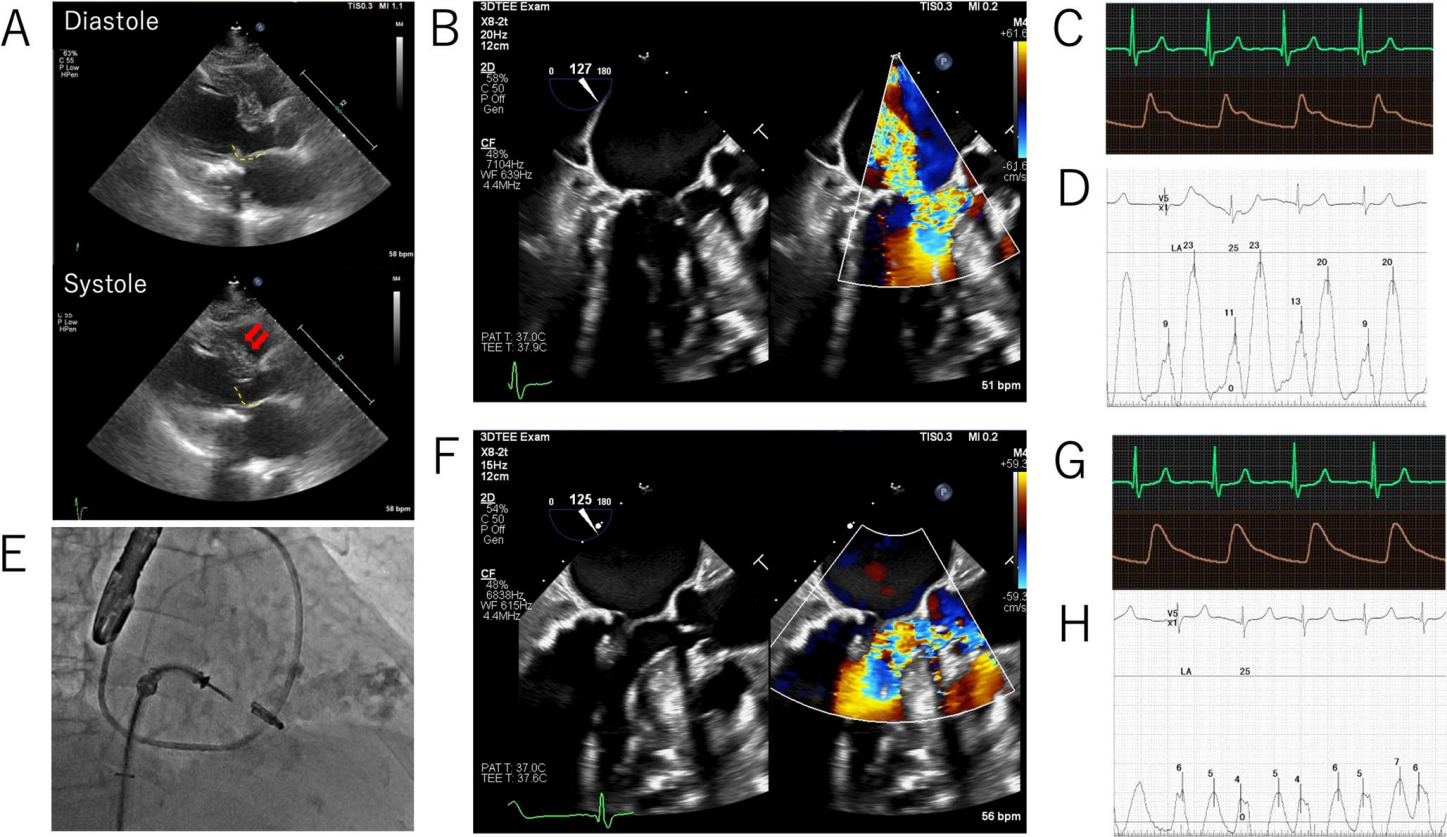
Imaging and hemodynamic changes before and after MitraClip implantation (**A**) Baseline transthoracic echocardiography showing sigmoid septum (arrow) and systolic anterior motion of the anterior mitral leaflet (yellow dotted line) with severe mitral regurgitation (MR) (**B**) Baseline transesophageal echocardiography demonstrates severe MR (**C**, **D**) Baseline hemodynamics: aortic and left atrial pressure waveform (**E**) Fluoroscopic image during clip positioning (**F**) Final transesophageal echocardiography showing trivial MR (**G**, **H**) Final hemodynamics: aortic and left atrial pressure waveforms

Hemodynamic tracings demonstrated aortic pressure waveform with spike-and-dome morphology (Fig. 1C) and a large left atrial v-wave (Fig. 1D).　Given prohibitive surgical risk, the Heart Team performed transcatheter edge-to-edge repair. A single MitraClip™ G4 NT device (Abbott Vascular, Santa Clara, CA, USA) was implanted at medial A2–P2 (Fig. 1E). Final TEE showed trivial MR and abolition of SAM (Fig. 1F). Aortic pressure waveform normalized (Fig. 1G), and the left atrial v-wave decreased (Fig. 1H). The mean transmitral gradient was 4 mmHg. The patient was discharged home on day 5 without complications.

SAM of the mitral valve is classically associated with hypertrophic obstructive cardiomyopathy but may also occur in elderly patients with a sigmoid septum, causing severe MR and dynamic LVOT obstruction. This case illustrates that MitraClip can be a valuable therapeutic option for SAM-related MR due to sigmoid septum, even in very elderly patients, when surgery is not feasible. M-TEER was selected only after multidisciplinary discussion when optimal medical therapy proved insufficient. Although current guidelines recommend discontinuing angiotensin receptor blocker therapy because of concerns about aggravation of LVOT obstruction, the medication was continued at a modest dose due to her prior ischemic stroke and marked systolic hypertension (>160 mmHg). Previous reports described late post-operative SAM with sigmoid septum [1]. Our case is the first in a 95-year-old with native-valve SAM-MR successfully treated with transcatheter edge-to-edge repair, with detailed hemodynamic correlations.

## Supplementary Information

Below is the link to the electronic supplementary material.

Supplementary material 1 (DOCX 398.6 kb)
