# Supplementary material for: Resolution of severe SAM-related mitral regurgitation in sigmoid septum by MitraClip in a 95-year-old woman
Source: Cardiovasc Interv Ther. 2025 Nov 28;41(2):476–7. doi: 10.1007/s12928-025-01219-1 (PMC13002665; doi:10.1007/s12928-025-01219-1)
Supplement: Supplementary file 1 — Supplementary material 1 (DOCX 398.6 kb) [file 12928_2025_1219_MOESM1_ESM.docx]

Supplementary Figure 1.

Supplementary Figure 1. Hemodynamic and imaging changes under provocation. (A) Transthoracic echocardiography continuous-wave (CW) Doppler of the left ventricular outflow tract (LVOT) at rest (≈2.0 m/s) and during Valsalva maneuver (>4 m/s). (B) Transesophageal echocardiography (TEE) CW Doppler of the LVOT at rest (2.7 m/s) and during handgrip exercise (>6 m/s). (C) TEE color Doppler showing mitral regurgitation (MR) severity at rest (trivial) and during handgrip exercise (severe MR due to systolic anterior motion).
